# Murine Embryonic Stem Cell Plasticity Is Regulated through *Klf5* and Maintained by Metalloproteinase MMP1 and Hypoxia

**DOI:** 10.1371/journal.pone.0146281

**Published:** 2016-01-05

**Authors:** Aya Abou Hammoud, Nina Kirstein, Virginie Mournetas, Anais Darracq, Sabine Broc, Camille Blanchard, Dana Zeineddine, Mohamad Mortada, Helene Boeuf

**Affiliations:** 1 Univ. Bordeaux, CIRID, UMR5164, F-33 000 Bordeaux, France; 2 CNRS, CIRID, UMR 5164, F-33 000 Bordeaux, France; 3 Lebanese University, Beyrouth, Liban; University of Kansas Medical Center, UNITED STATES

## Abstract

Mouse embryonic stem cells (mESCs) are expanded and maintained pluripotent *in vitro* in the presence of leukemia inhibitory factor (LIF), an IL6 cytokine family member which displays pleiotropic functions, depending on both cell maturity and cell type. LIF withdrawal leads to heterogeneous differentiation of mESCs with a proportion of the differentiated cells apoptosising. During LIF withdrawal, cells sequentially enter a reversible and irreversible phase of differentiation during which LIF addition induces different effects. However the regulators and effectors of LIF–mediated reprogramming are poorly understood. By employing a LIF-dependent ‘plasticity’ test, that we set up, we show that *Klf5*, but not *JunB* is a key LIF effector. Furthermore PI3K signaling, required for the maintenance of mESC pluripotency, has no effect on mESC plasticity while displaying a major role in committed cells by stimulating expression of the mesodermal marker Brachyury at the expense of endoderm and neuroectoderm lineage markers. We also show that the MMP1 metalloproteinase, which can replace LIF for maintenance of pluripotency, mimics LIF in the plasticity window, but less efficiently. Finally, we demonstrate that mESCs maintain plasticity and pluripotency potentials *in vitro* under hypoxic/physioxic growth conditions at 3% O_2_ despite lower levels of *Pluri* and *Master gene* expression in comparison to 20% O_2_.

## Introduction

During the last decades, mouse embryonic stem cells (mESCs) have been intensively studied to reveal genetic programs essential for control of pluripotency and early cell fate decisions. This led to the characterization of signaling pathways and transcription effectors essential for the maintenance of mESCs pluripotency. These include the leukemia inhibitory factor (LIF)/signal transducer and activator of transcription 3 (STAT3)/suppressor of cytokine signaling 3 (SOCS3) pathway, along with the ‘*Master* genes’ like Octamer 4 (*Oct4*), *Nanog*, SRY (sex determining region Y)-box 2 (*Sox2*) and estrogen-related receptor, beta (*Essrb)* [1–3]. Subsequently, cocktails of genes were identified that could drive reprograming of many types of somatic cells (like fibroblasts, keratinocytes, hepatocytes or bone marrow-derived cells), from various species including Humans, to induced pluripotent stem cells (iPSCs), with potential applications in cell therapies and regenerative medicine [4–6].

The mESCs are derived from pre-implantation blastocysts and are maintained pluripotent *in vitro* in i) serum-containing medium with LIF, or ii) bone morphogenetic protein 4 (BMP4)/LIF medium, or iii) serum-free medium supplemented with LIF and cocktails of inhibitors for key signaling pathways [extracellular regulated kinase (ERK), fibroblast growth factor (FGF) and glycogen synthase kinase 3β (GSK3β) inhibitors, 3i]. All these cell growth media maintain mESCs in a naive pluripotent state, the most immature *in vitro* state defined by the cells being capable of colonizing embryos and contributing to all cell types in the organism [7–10]. Human embryonic stem cells (hESCs), which are maintained pluripotent in the presence of FGF2 and activin A are closer to primed mouse epiblast stem cells (EpiSCs), a state more prone to differentiation and less stable than the naive state. However various studies have reported strategies to revert hESCs to a naive state by treatment with LIF, STAT3 and/or signaling pathway inhibitors [11–14].

The LIF-induced signaling cascade starts with activation of Janus kinase (JAK) phosphorylating phosphatidylinositol 3-kinase (PI3K), which induces the phosphorylation and activation of AKT serine/threonine kinase. AKT signaling leads to the activation of T-box 3 (*Tbx3*) expression, a transcription factor that induces the expression of pluripotency maintaining genes. Additionally, AKT inactivates GSK3β by phosphorylation preventing the ubiquitin-dependent degradation of Myc and inhibition of *Nanog* expression. GSK3β is also inhibited by the canonical wingless (Wnt) signaling pathway which acts in synergy with LIF to maintain the expression of pluripotency related genes [15–18].

Most stem cells are found in complex microenvironments, termed ‘niches’ which reside in low oxygen concentration ([O_2_]), [19,20]. mESCs are derived from embryos which also remain in 1.5–5% [O2]. This low oxygen environment is physiologically normal, not only for ESCs but also for many other types of stem cells including neural stem cells (NSCs), hematopoietic stem cells (HSCs) and mesenchymal stem cells (MSCs) [21–23]. The effect of low [O_2_] on ESCs fates remains controversial and poorly understood. Several reports have shown that low [O_2_] inhibits differentiation and maintains pluripotency of hESCs [24–27] and improves clonal survival of mESCs, particularly when the GSK3β pathway is repressed [7,28,29]. Also the Wnt/ b-Catenin pathway is stimulated, under hypoxia in mESCs which could be differentiated into the three cell lineages *in vitro*, indicating that hypoxia did not alter their pluripotency [30,31]. Activation of the Notch signaling pathway by hypoxia stabilizes hypoxia induced factor 1α (Hif1α), the major effector of early steps of hypoxia, maintains the undifferentiated state and thus enhances the generation of both human and mouse iPSCs [32]. In addition, hypoxia can also revert hESCs or iPSC-derived differentiated cells back to the stem cell-like state [33]. In contrast, other studies reported that low [O_2_] facilitates the differentiation of mESCs by impairing LIF signaling [34]. In addition, hypoxia enhances differentiation of hESCs towards cardiomyocytes and chondrocytes [35,36] and stimulates vascular differentiation through HIF1α-mediated repression of *Oct4* and activation of vascular endothelial growth factor (*Vegf*) [37].

Recently, the metalloproteinase MMP1, shown to degrade the extracellular matrix (ECM) of mESCs, came as another microenvironment component essential for maintenance of mESC pluripotency. Indeed, it was shown that MMP1, either secreted by feeder cells or added to cell culture medium could release the ECM-trapped ciliary neurotrophic factor (CNTF) cytokine which then activates the JAK/STAT3 pathway, as does LIF [38,39].

By various microarray analyses performed on pluripotent versus differentiated cells, the core pluripotency genes were characterized by specific knockdown strategies [40,41]. In such analysis, we defined a cluster of *Pluri* genes [including muscle and microspikes RAS (*Mras*), *Esrrb* and T cell lymphoma breakpoint 1 (*Tcl1*)] whose expression diminishes within the first day (d1) of LIF removal. Among these genes a particular subset, involved in cell adhesion and cell-cell contacts, had also been characterized (including carcinoembryonic antigen-related cell adhesion molecule 1 (*Ceacam1*), gap junction protein beta 3 (*Gjb3*), gap junction protein beta 5 (*Gjb5*) and junction adhesion molecule 2 (*Jam2*). Furthermore, a cluster of early differentiation genes, named *Diff*, is induced starting d1 of LIF withdrawal (including lymphoid enhancer binding factor 1 (*Lef1*), *carbonic anhydrase 4* (*Car4*) and protocadherin 1 **(***Pcdh1*), [42]. In addition, we identified LIF-induced (*Lifind*) genes, whose expression is stimulated by very short LIF treatment after a period of LIF withdrawal of 1 or 2 days. There are two categories of *Lifind* genes: those expressed after LIF induction specifically in cells depleted of LIF for 24 hours (*speLifind)* like Kruppel-like factor 5 (*Klf5*) and those induced in a more general way, in immature as well as differentiated cells (*pleioLifind*) like *JunB*. [42–44]. *Klf5*, a transcription factor of the Kruppel gene family, well conserved upon evolution, has been shown to be essential for the derivation of mESCs from Inner Cell Mass (ICM) as well as for the maintenance of mESCs cell pluripotency *in vitro* [45–48]. *Klf5*^*-/-*^ ES cells differentiates spontaneously at high frequency and in knock-down experiments an essential effect of *Klf5* to block specific mesodermal differentiation has been demonstrated. In reverse experiments it has been shown that overexpression of *Klf5* leads to LIF-independent self-renewal with high level of *Tcl1* expression and increased cell proliferation along with AKT phosphorylation and stimulation [45,46,49,50].

By studying early steps of differentiation initiated by LIF withdrawal, we have previously shown that cells enter commitment phases during which LIF plays differential roles. While at d1 of LIF depletion re-addition of LIF blocks differentiation and/or reverts cells towards a pluripotency state, at d2 and later, re-addition of LIF is less efficient in altering the differentiation process and blocking apoptosis [43,51]. To understand the molecular mechanisms involved in the regulation of this early LIF-dependent mESC plasticity we set up an *in vitro* test for studying the impact of genes and signaling pathways which could modulate these properties in mESCs. In addition, we analysed the effects of MMP1, a new stemness player, and of hypoxia in mESC plasticity. We discovered that *Klf5* but not *JunB* is acting on cell plasticity. We also show that MMP1 can replace LIF to a certain extent in the plasticity test and that mESCs remain plastic under hypoxic conditions, despite alterations in gene expression patterns.

## Materials and Methods

### Cell culture

The mESC line CGR8 (from A. Smith laboratory) was grown in 6 well plates coated with 0.2% gelatin under 7% CO_2_ humidified atmosphere at 37°C in Dulbecco’s Modified Eagle’s Medium, Sigma Aldrich (DMEM-glutamax) supplemented with 10% fetal calf serum (FCS, Dutscher) or 10% KnockOut™ Serum Replacement (SR), (Gibco), 0.1 mM β-mercaptoethanol (Sigma-Aldrich), 400 μg/mL gentamicin (Gibco Invitrogen) in the presence of 10 ng/mL of human LIF. Cells were passaged every 2 or 3 days by trypsinization and resuspended in complete medium.

For experiments performed under hypoxia (3% O2), cultures were performed in cell chamber with O2 and CO2 regulators (BioSpherix) under water saturated atmosphere.

### Embryoid bodies and differentiation procedures

mESCs were grown in bacterial Petri-dishes in DMEM glutamax supplemented with 10% SR for 8 days under normoxia or hypoxia (BioSpherix cell chamber). At d8, embryoid bodies (EBs) were plated in gelatin-coated flasks for 7 days under normoxia or hypoxia. Beating cardiomyocytes were detected the day after plating and the neuronal cells with dense networks were observed from 3 days after plating.

### Drug treatment (LY294002)

The analysis of the effect of the PI3K inhibitor LY294002 (Cell signaling) was done by pre-treating the cells with 50 μM LY294002 during 1h before treatment with LIF. Then LIF induction was performed for 1 to 4 days in the presence of the drug added for the first 24h. The efficiency and specificity of the inhibitor was verified by Western blot analysis on Phospho-AKT (anti-Phospho-ser473 AKT from Cell signaling, data not shown).

### MMP1 treatment

Cells grown in complete medium were washed with PBS and incubated in medium without LIF in the presence of 100 ng/mL of recombinant human metalloproteinase 1 (MMP1; cat. 420–01, PeproTech). Medium was changed every day.

### Small interfering RNA transfection

Cells were depleted from antibiotics 24h before transfection. 20 nM of siRNAs targeting *Klf5* or *JunB* (ON-TARGET*plus* SmartPOOL (Dharmacon), and 5 μL Lipofectamine 2000 (Invitrogen) were incubated separately in 250 μL DMEM glutamax for 5 min at room temperature. Then, both solutions were gently mixed and incubated for another 20 min before being added to cell culture. As a control, the same concentration of siRNA [ON-TARGET*plus* Non-targeting Pool (Dharmacon)] was used. SiRNA transfection was done on about 5.10^5^ cells in suspension in cell medium without antibiotics. The medium was changed 24h after transfection and medium with LIF was added for 3 days. To determine siRNA efficiency at the beginning of the induction process, the expression of each target gene was verified by RT-qPCR analysis after 1h of LIF induction. The knock-down was considered to be efficient when gene expression during LIF induction equals the level of expression in non-induced cells, which was the case for both studied genes (data not shown).

### RNA isolation and quantitative real-time PCR

For RNA isolation, 800 μL of TRIzol reagent (Invitrogen) were added to the cells and RNAs were prepared according to the manufacturer’s instructions. The reverse transcriptase (RT)/DNase step was performed with the QuantiTect Rev Transcription kit (Qiagen). Quantitative real time (q)-PCR was performed using the Applied Biosystems StepOne^TM^ Real-Time PCR System in a 25 μL reaction volume containing 5 μL of cDNA (1:20 dilution of the reverse transcribed sample), 12.5 μL of B-R SYBR^®^ Green SuperMix for iQ (Quanta, BioSciences) and primers pair at a final concentration of 0.5 μM. The PCR program included a denaturation step at 95°C for 10 min, an amplification step for 40 cycles (15 s at 95°C, 1 min at 60°C) and a final dissociation curve step in order to determine the specificity of the product. Samples were duplicated for each run. Quantification was done by calculating the 2^∆∆Ct^ value. Data were normalized using the *Hprt* mRNA, known to remain constant in the experimental condition.

Statistical significance of differential gene expression was calculated from four or more independent experiments by using IBM SPSS Statistics 21 software.

### Protein cell lysates and Western blot

Cells were lysed in mild RIPA buffer [PBS 1x, 1% Triton-X-100 (Sigma), 1% NP-40 (Sigma), 0.05% SDS] supplemented with protease inhibitor cocktail (Sigma), 1 mM Pefabloc (Sigma), 40μM NAF, 1mM Na3VO4 and centrifuged for 20 min at 15 000 g. Antibodies were diluted 1:1000 as follows: rabbit polyclonal anti-gp190 (Novus, NBP2-32070); rabbit monoclonal anti-Phospho-tyr705 STAT3 (D3A7, Cell Signaling); rabbit monoclonal Anti-STAT3 (ABCAM, Ab68153); rabbit polyclonal Anti-Oct4 (ABCAM, Ab18976); rabbit polyclonal anti-Nanog (ABCAM, Ab80892); goat polyclonal anti-Sox2 (Santa Cruz, sc-17320); Rabbit polyclonal Anti-Erk2 (Santa Cruz, sc-154). Primary antibody incubation was done overnight in a sealed plastic bag at 4°C with slight agitation in the Odyssey blocking buffer (LI-COR company). After washing with TBS 0,1% Tween (twice for 10 min), the membrane was incubated for 1 h with the fluorescent far-red coupled secondary antibody, in accordance with the primary antibody: IRDye 680RD goat (polyclonal) anti-rabbit IgG (H+L), (LI-COR) or IRDye 800RD goat (polyclonal) anti-mouse IgG (H+L), (LI-COR) or HRP-labeled anti-Goat (IgG (H+L) (Vector Laboratories), diluted 1:20 000 in Odyssey blocking buffer. After washing twice for 10 min with TBS 0.1% Tween and with TBS1x, membranes were revealed with the Odyssey^FC^ (LI-COR) apparatus with the Image studio software as recommended by manufacturer. Quantification of the correct size band for each antibody was performed with the Odyssey^FC^ (LI-COR) quantification image studio software.

### Flow cytometry

**Fig 1:**
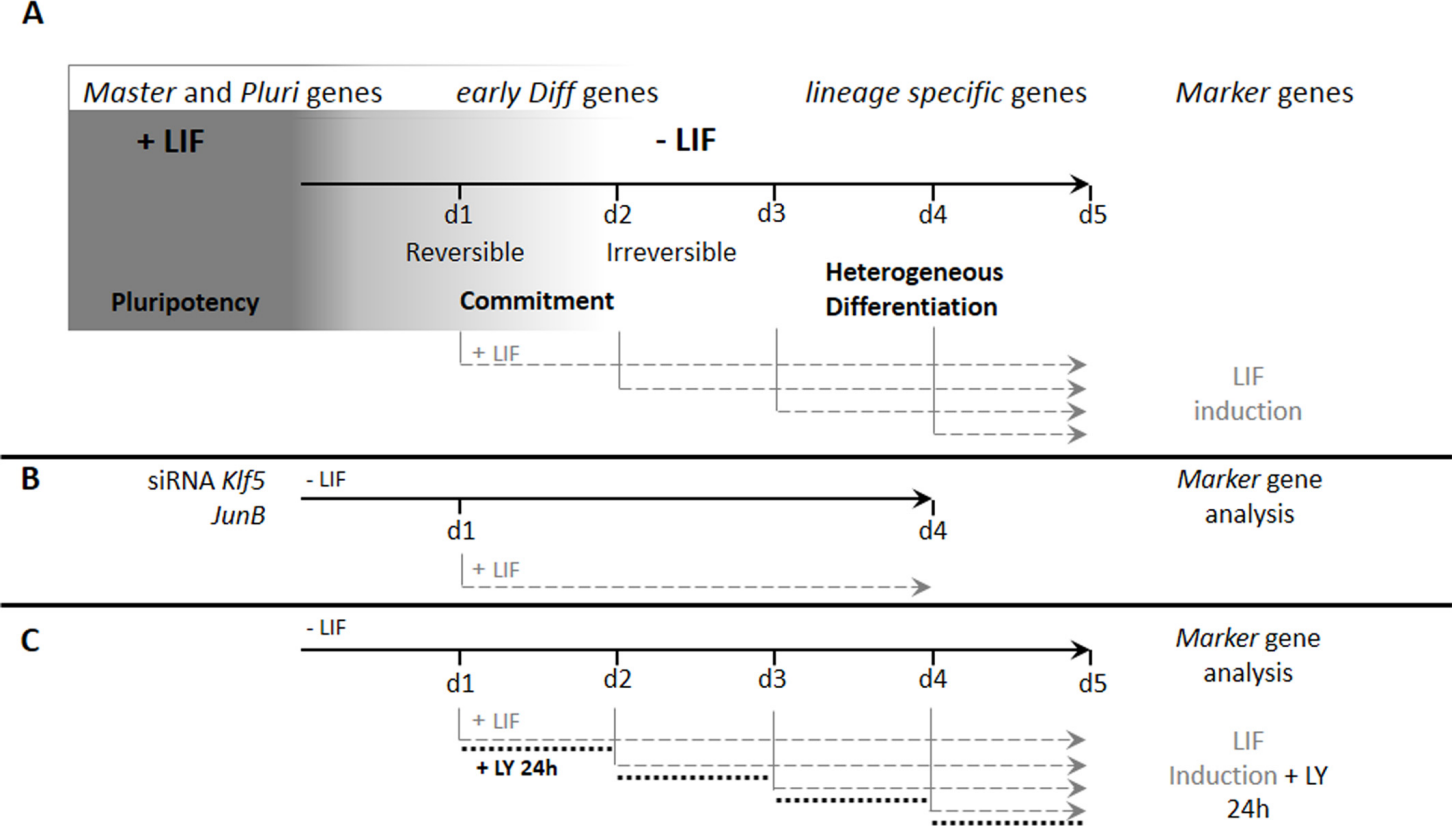
General scheme of the ‘mESC Plasticity Test’. (A) mESCs maintained in pluripotency (+LIF) or cultivated without LIF for the indicated period of time in days (d1 to d5) are induced with LIF at different time points. (B) The effect of specific genes was tested by a siRNA strategy in which addition of siRNA was performed the first day of LIF withdrawal. (C) PI3K inhibitor LY was added for 24 hours to the committed or differentiated cells as indicated.

mESCs were grown in the presence or absence of LIF with or without LIF re-addition as indicated, as described in the Fig 1, on a kinetic of 4 or 5 days. At the end point of the experiment, cells were trypsinized and the centrifugated pellets were washed in PBS 1X. 100 000 cells of each point were distributed in 96 round well plates. Cells were incubated in 50 μL of primary anti-Ceacam1-Phycoerythrin antibody (Biolegend, BLE 134506, diluted 1/10), in PBS 1X, 0.5% BSA, 1mM EDTA for 30 min. at 4°C in dark. Cells were then washed twice with 250 μL of PBS 1X and the resuspended pellets in PBS 1X were transferred in specific flow cytometry tubes and processed on the Fortessa cytometer (Becton Dickinson). Non labelled and isotype labelled cells were used as negative control. The percentage of positive cells and the MFI were obtained with the DIVA software BD Biosciences. The overlay “half off set graph” was displayed with the gated P1 population, with the Flow Jo software.

## Results

### Set up of a ‘Plasticity test’ in the mESC model

To characterize the behavior of mESCs during the first days of LIF withdrawal and to precisely determine how cells respond to LIF during the commitment phases, we set up an *in vitro* assay, hereafter referred to the ‘plasticity test’. In this test, cells were stimulated to differentiate by simple LIF withdrawal for 1 to 4 or 5 days and then LIF was reintroduced at different time points as depicted in Fig 1A. Analysis of gene expression by RT-qPCR was performed at each time point of the experiment. This assay enabled us to study the effects of key genes by siRNA depletion as depicted in Fig 1B, or signaling pathways by the use of chemical inhibitors, as depicted in Fig 1C.

**Fig 2:**
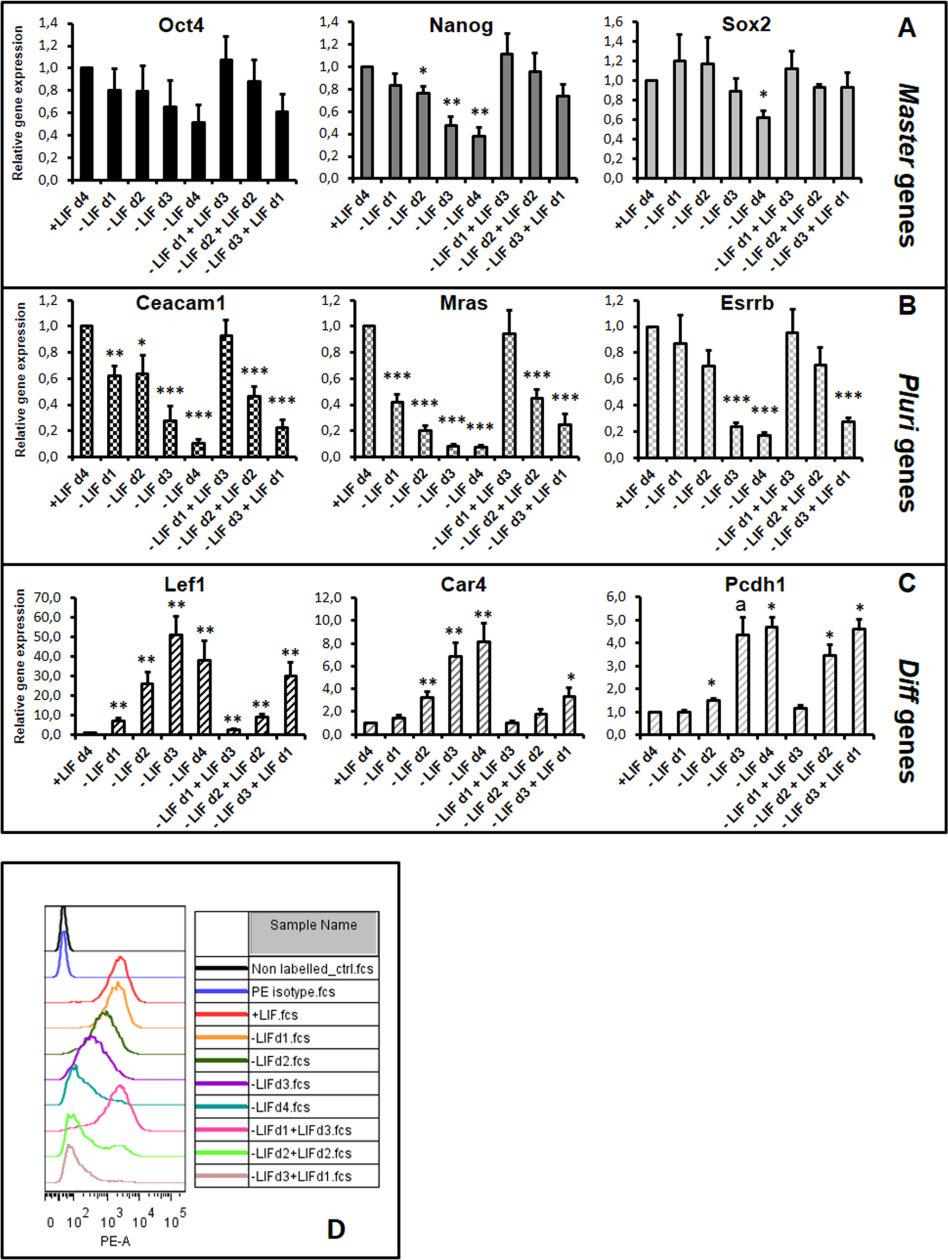
LIF has differential effects on mESC-derived cells, after d1, d2 or d3 of LIF starvation. Cells were depleted from LIF or induced with LIF after a period of LIF depletion as indicated. After 4 days, expression of the selected genes (A) *Master* genes, (B) *Pluri* genes and (C) *Diff* genes was analyzed by RT-qPCR with *Hprt* used for normalization. Graphs represent the average level of expression and standard error of the mean (SEM) bars, calculated from 4 independent experiments. +LIF condition was arbitrarily set as 1. One sample t-test was performed for each condition versus the +LIF sample: *p-value<0.05; **p-value<0.01, ***p-value<0.001; a: p-value = 0.051; if not stated: not significant. (D) A representative experiment of flow cytometry, performed in the indicated conditions with the Ceacam-PE antibody, is presented: "off set graph" of the MFI of the total P1 gated cell population of each cell growth condition, with the Flo Jo software.

In this assay, we analyzed the expression profiles of the *Master* genes (*Oct4*, *Sox2* and *Nanog*) and of the newly identified *Pluri* (*Ceacam1*, *Mras* and *Esrrb*) and *Diff* (*Lef1*, *Car4* and *Pcdh1*) genes (Fig 2). The expression level of *Master* genes was not strongly regulated during the first days of LIF withdrawal as expected [42,52]. These genes were also highly expressed after LIF reintroduction, up to d3 (Fig 2A). In contrast, the expression level of *Pluri* genes (known to decrease after one day of LIF withdrawal [42]), was re-established by LIF treatment at d1 but not restored or less efficiently re-established at d2 and d3 (Fig 2B). In addition, the expression level of the examined *Diff* genes was also regulated: their expression was stimulated upon LIF withdrawal and repressed upon LIF treatment at d1 but less efficiently at d2 and d3 (Fig 2C). Interestingly, the LIF effect observed after 4 days [(-LIFd1+LIFd3), Fig 2] started shortly after LIF addition [within the first 24h, (-LIFd1+LIFd1), S1 Fig], ruling out a selection process of LIF-resistant cells, enriched after 4 days of culture.

In addition to gene expression analysis by RT-qPCR we also analyzed endogenous protein expression by flow cytometry with an antibody raised against the membrane-associated protein Ceacam1 (encoded by the *Ceacam1* Pluri gene). Mean of Fluorescence Intensity (MFI) analysis of the bulk population (gated P1 cell population) revealed a global decrease of Ceacam1 expression, starting at d1 of LIF withdrawal with a continuous loss until the end of LIF kinetic starvation. Expression is regained at the -LIFd1+LIFd3 condition but not at–LIFd2+LIFd2, which follows the RNA profile (Fig 2D). This experiment indicates that, upon LIF withdrawal, there are no detectable resistant cells expressing higher level of Ceacam1 protein which could have remained and should be observed as a double peak in such analysis.

### The Klf5 gene is involved in the regulation of the plasticity window

**Fig 3:**
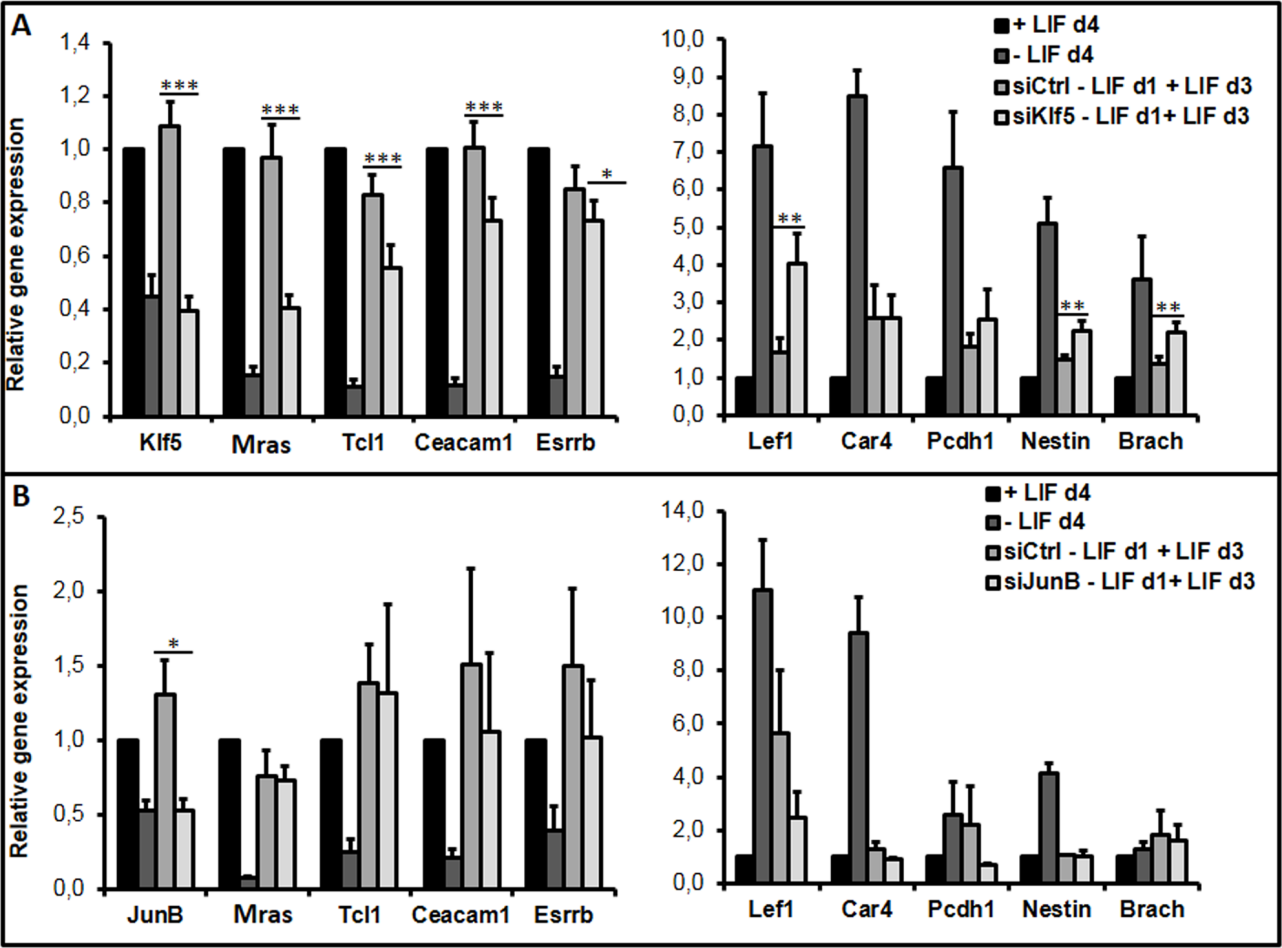
*Klf5* but not *JunB* disturbs the LIF-dependent plasticity in early committed cells. Cells were depleted from LIF for 24 hours and either transfected with siControl (siCtrl) (A) siKlf5, or (B) siJunB and then induced with LIF for three days (d3). Graphs represent the average level of expression and SEM from 3 to 6 independent experiments. Paired sample t-test was performed to determine the significance of the difference in the expression levels observed with the non specific (siCtrl) versus specific siRNA: *p-value<0.05; **p-value<0.01, ***p-value<0.001; if not stated: not significant.

We previously identified two groups of genes induced by LIF (*speLifind* and *pleioLifind* genes) and postulated that some of the *speLifind* genes, induced by LIF preferentially at d1 but less at d2 after LIF withdrawal, could be involved in such reversion process [43]. As a proof of principle, by using siRNA strategy, we analyzed the impact of *Klf5* (*speLifind*) and *JunB* (*pleioLifind*) knockdown on cell plasticity as described in Fig 1B. The down-regulation of *Klf5* during LIF induction led to specific repression of the *Pluri* genes and induction of early *Diff* markers like *Lef1*, *Nestin and Brachyury (Brach)* (Fig 3A). In contrast, no effect on *Pluri* or *Diff* gene expression was detected with *JunB* siRNA, although it efficiently repressed JunB expression (repression was 100% in the first hours of LIF induction and was at least of 50% after 4 days, at the end point of the experiment, (data not shown and Fig 3B). These results indicate that *Klf5* is involved in the LIF response at d1 while *JunB* does not regulate this process.

### PI3K does not regulate early commitment but it acts later for cell fate choices

**Fig 4:**
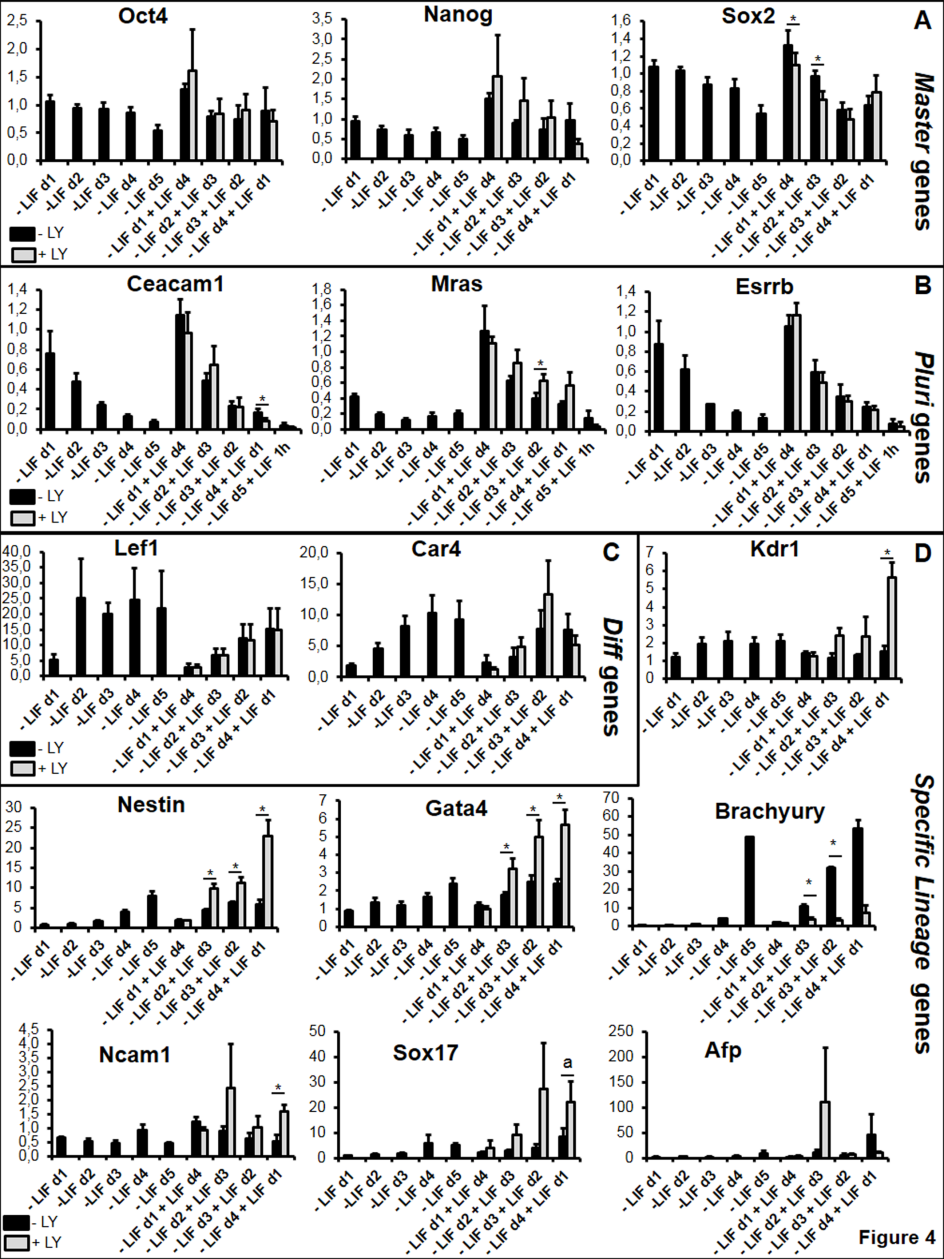
PI3K does not regulate the LIF-dependent plasticity window but stimulates differentiation towards early mesoderm lineage while inhibiting ectoderm and endoderm differentiation processes. Cells were depleted from LIF or induced with LIF in the presence of LY, for the first 24 hours as described in Fig 1C. After 5 days, expression of the selected genes (A) *Master* genes, (B) *Pluri* genes, (C) early *Diff* genes and (D) specific germ layer genes, was analyzed by RT-qPCR. Graphs represent the average level of expression and SEM from 4 independent experiments. Paired sample t-test was performed to evaluate the significance of the difference in the expression levels observed with or without LY: *p-value<0.05; **p-value<0.01, ***p-value<0.001; a: pvalue = 0.051; if not stated: not significant.

LIF induces the PI3K pathway allowing the maintenance of mESCs pluripotency [15,16,52]. We investigated the effect of a specific PI3K inhibitor (LY294002 abbreviated LY) in the ‘plasticity test’ as described in Fig 1C. We analyzed gene expression at the end point of the kinetics (5 days) in the presence or absence of LY, added for 24 hours in cell medium at each point of the kinetics. PI3K inhibition had faint effects on the *Sox2* master gene while displaying no effects neither on *Oct4* nor on *Nanog*, *Pluri* and *early Diff* genes indicating that PI3K is not strongly involved in LIF-dependent cell plasticity (Fig 4A–4C). However, addition of the inhibitor at later time points (at d3 and d4 upon LIF withdrawal) led to changes in the expression of some early lineage markers: while expression of *Nestin* and to less extend of Ncam1 (ectoderm marker) and of *Gata4* (endoderm marker) was induced, the expression of *Brachyury* (early mesoderm marker) but not of *Kdr1* (late mesoderm marker) was strongly repressed. Thus, active PI3K seems to repress ectoderm and endoderm while inducing mesoderm during early onset of differentiation (Fig 4D).

### Slight variations in cell responses in Serum versus SR-containing medium

Since the behavior of mESCs can fluctuate depending on serum batches which have various effects on cell proliferation and pluripotency ([53] and our unpublished results), we also performed the ‘plasticity test’ in medium supplemented with the serum replacement (SR) compound instead of foetal calf serum. While similar results were found for *Pluri* gene expression (like *Ceacam1*, *Mras* and *Esrrb*), we observed that the plasticity window was slightly delayed with an effect of LIF extended up to d2 for *Diff* genes (S2 Fig). To reduce serum-side effects in our experiments, we performed all the following assays in medium supplemented with 10% SR rather than in medium containing serum.

### MMP1 is a novel stemness factor that regulates the plasticity window

**Fig 5:**
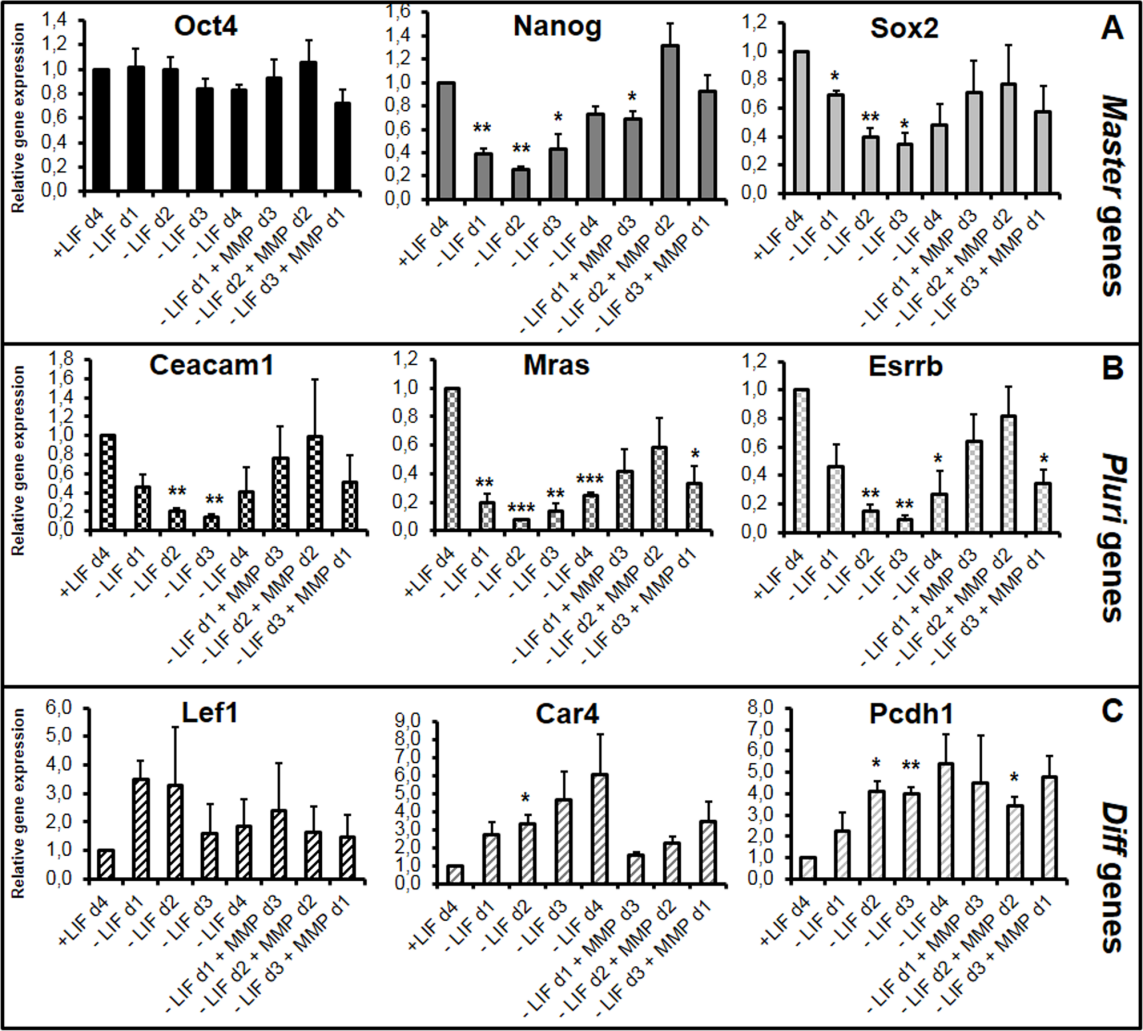
MMP1 metalloproteinase partly mimics the LIF effect. Cells were depleted from LIF for the indicated time and induced with 100 ng/mL of purified MMP1 as indicated. After 4 days, expression of the selected genes (A) *Master* genes, (B) *Pluri* genes and (C) *Diff* genes was analyzed by RT-qPCR. Graphs represent the average level of expression and SEM as depicted in Fig 2. One sample t-test was performed for each condition *versus* the +LIF sample: *p-value<0.05; **p-value<0.01, ***p-value<0.001; if not stated: not significant.

We analyzed the impact of the MMP1 metalloproteinase, recently shown to be secreted by feeder cells and to degrade the extracellular matrix (ECM) of mESCs leading to the release of the ECM-trapped ciliary neurotrophic factor (CNTF) cytokine [39]. This LIF family member displays similar effects to LIF on mESCs, and maintains mESC pluripotency by inducing the JAK1/ STAT3 pathway [39]. In our assay, we replaced LIF by MMP1 and observed cells remaining pluripotent for up to 11 days, as previously shown ([39] and our data not shown). In the ‘plasticity test’, MMP1 mimicked the LIF effect, however, with a less potent effect on the expression of *Diff* genes (Fig 5). Nevertheless, this experiment showed that MMP1-dependent remodeling of the extracellular matrix is involved in cell plasticity.

### Hypoxia maintains pluripotency and plasticity *in vitro* despite gene expression changes versus normoxia

**Fig 6:**
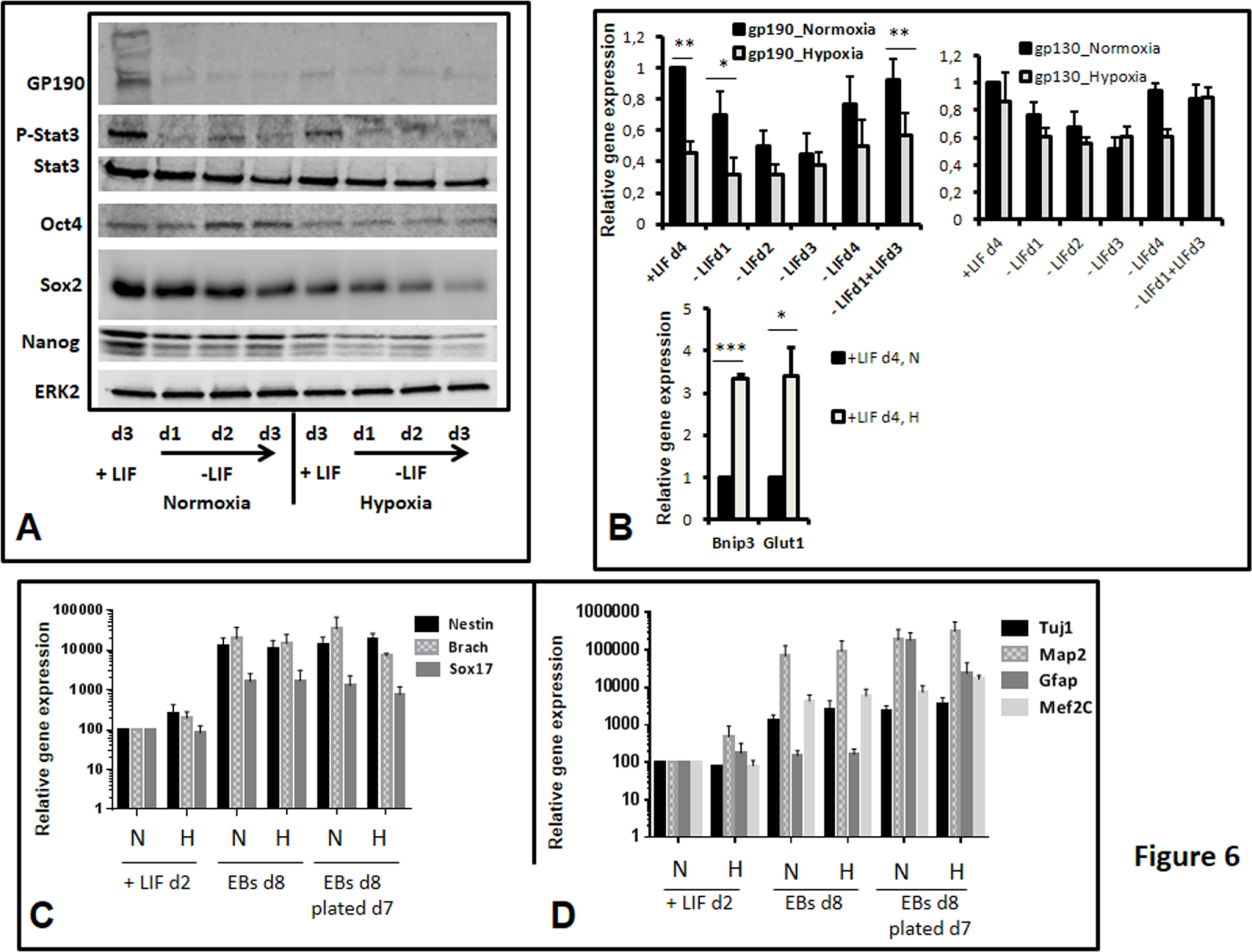
mESCs grown under 3% O2 have an impaired LIF signaling, express less ‘pluripotency-involved’ proteins but maintain pluripotency. Cells were grown with (+LIF) or without LIF for 1 to 3 or 4 days under normoxia or hypoxia as indicated. (A) Protein cell lysates (60 μg) from cells grown under the indicated conditions were analyzed by Western blot. ERK2 was used as a loading control. A representative experiment is shown. Quantification of signals obtained with the different antibodies in the + LIF condition (n = 4) is provided in S3 Fig (B) Gene expression of the indicated genes was analyzed by RT-qPCR. Graphs represent the average level of expression and SEM as depicted in Fig 2 (n = 4). Paired sample t-test was performed to evaluate the significance of the difference in the expression levels observed in normoxia versus hypoxia: *p-value<0.05; **p-value<0.01, ***p-value<0.001; if not stated: not significant. (C) early lineage markers, (D) specific lineage markers in mESCs induced for *in vitro* differentiation under normoxia (20% O2, N) or hypoxia (3% O2, H). Gene expression was analyzed by RT-qPCR in cells grown for two days under N or H conditions (+LIFd2), in EBs at day 8 of their formation (EBs d8) and seven days after EBs plating. Y axis is in log scale.

**Fig 7:**
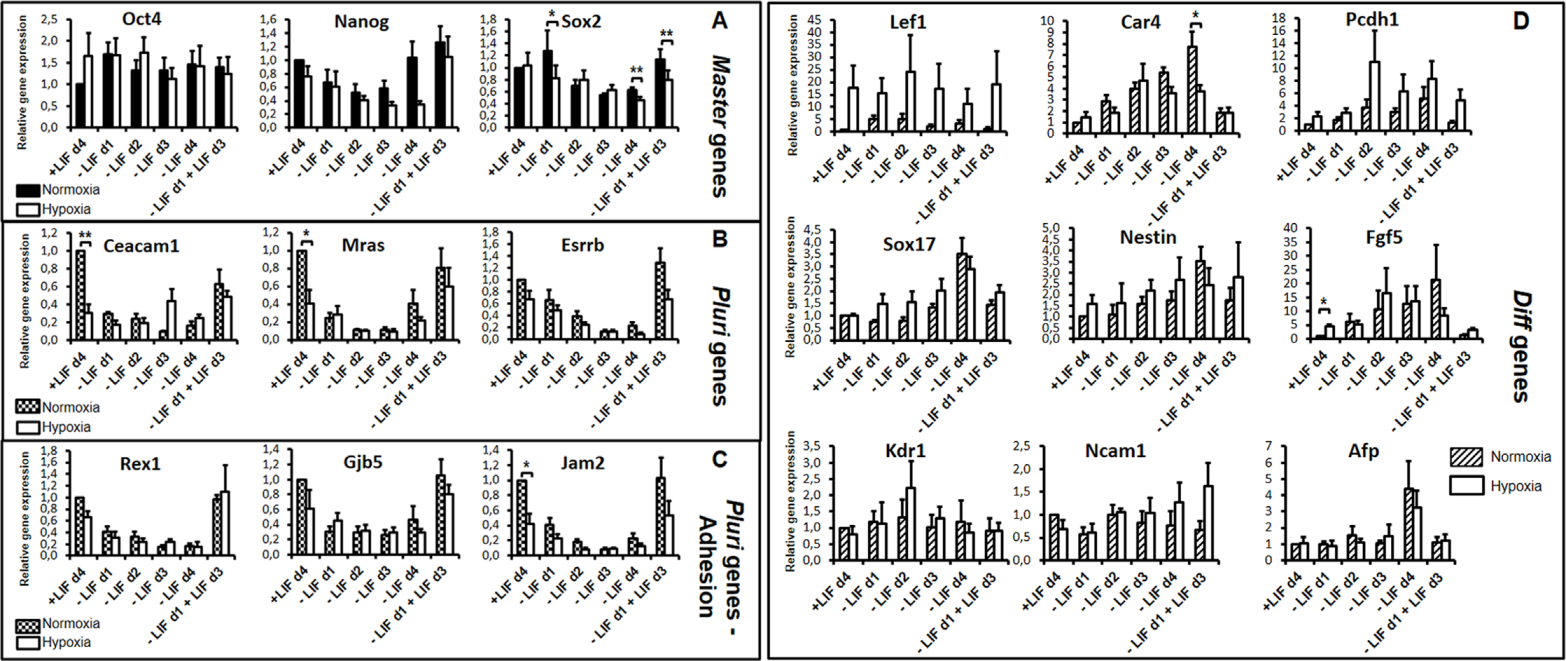
Hypoxia maintains cell plasticity with a novel equilibrium of *Pluri* and *early Diff* genes. Cells grown under normoxia (N) or hypoxia (H) were depleted from LIF or induced with LIF after a period of LIF depletion as indicated. After 4 days, expression of the selected genes, (A) *Master*, (B) *Pluri*, (C) *Pluri Adhesion* and (D) *Diff* was analysed by RT-qPCR. Graphs represent the average level of expression and SEM as depicted in Fig 2 (n = 4). One sample t-test was performed for the +LIF condition and paired sample t-test was performed for the other conditions to evaluate the significance of the difference in the expression levels observed in normoxia versus hypoxia: *p-value<0.05; **p-value<0.01, ***p-value<0.001; if not stated: not significant.

We also analyzed the potential plasticity of mESCs grown under low O_2_ (3%), which corresponds to physioxic (physiologic *in vivo*) conditions of pre-implantation embryos [7,54–57]. Cells were incubated at 3% O_2_ in a dedicated hypoxic cell chamber and incubator with cell medium changed in the hypoxia chamber without normoxic shock during the entire experiment. Under continuous 3% O_2_, protein expression levels of the LIF receptor subunit (gp190) and of Phospho-tyr705-STAT3 were lower than under normoxia indicating an impairment of LIF signaling in these conditions as previously shown (Fig 6A, S3 Fig and [34]). In addition, we showed that the expression levels of Oct4, Sox2 and Nanog proteins were decreased upon hypoxia (Fig 6A and S3 Fig). In addition, protein expression levels and STAT3 phosphorylation decrease upon LIF withdrawal under normoxia (unless for Oct4), as expected, but also under hypoxia. This attests that LIF signalling is activated, even if weaker under hypoxia. Hypoxia also induced the expression of Hif1α protein targets, like the BCL2/adenovirus E1B interacting protein 3 (*Bnip3*) and glucose transporter 1 (*Glut1*) genes, attesting that the Hif1α protein was stabilized and active (Fig 6B). However, despite delayed cell growth compared to 20% O_2_, as previously observed [34], cells in 3% O_2_ remained pluripotent as shown by morphological criteria (alkaline phosphatase staining, S4 Fig) and by their capacity to form embryoid bodies (EBs) and to differentiate into neurons and beating cardiomyocytes (data not shown). Analysis of expression levels of the three germ layer markers in our differentiation procedure (eight day-formed EBs followed by seven days of plated EBs) showed their efficient expression in both normoxia and hypoxia (Fig 6C). In addition, specific markers [neuronal like tubulin β3 (*Tubb3*/*Tuj1*) and microtubule associated protein 2 (*Map2*), glial like glial fibrillary acidic protein (*Gfap*) and cardiac like myocyte enhancer factor 2C (*Mef2c*)] were highly expressed under hypoxia with comparable levels to the expression under normoxia highlighting the pluripotent state of the starting cells. Interestingly, we show that cells kept their plasticity potential under 3% O_2_ conditions (Fig 7), despite lower Oct4, Nanog and Sox2 protein expression (Fig 6A and S3 Fig), lower expression of the *Pluri* genes (Fig 7B) and higher expression of *Lef1* and *Fgf5* in 3% O_2_ compared to 20% O_2_ (Fig 7D). Collectively, these results indicate that a novel equilibrium of gene expression and of post-translational regulation (as observed for Oct4, Nanog and Sox2) are established under low O_2_ in pluripotent cells. However, the expression levels of lineage markers (SRY (sex determining region Y)-box 17 (*Sox17*), *Nestin*, *Kdr1*, *Ncam1* and *Afp* (Fig 7D) and also *Brachuyry*, (data not shown) remained low in + LIF condition under 3% as well as 20% O_2_ concentration, emphasizing the point that cells do not differentiate in the presence of LIF, under low O_2,_ at least for the time in culture used here, up to four days.

## Discussion

Plasticity, which is a hallmark of stem cells, is defined in this study as the potential of committed cells to revert to a more immature state when grown in a particular environment. The characterization of regulators of this process is of major interest. For example, understanding the mechanism of the reversion of primed ESCs to the more immature naive state, is an essential step for future development of cellular therapies. Indeed, human ESCs or iPSCs are closer to primed than to naive states. Primed mouse EpiSCs can be reverted to the naive state of mESCs by exposure to LIF–STAT3 signaling, especially when cultured in the presence of signaling pathway inhibitors (*e*.*g*. 2i) or during transient expression of kruppel-like factor 4 (Klf4) or the myelocytomatosis oncogene (Myc) [58,59]. In Humans, the reversion of the naturally primed hESCs to naive state also involves the LIF-/STAT3 signaling pathway [11,12,14].

Here, we developed an *in vitro* assay showing that mESCs are equipped with a time-dependent window of plasticity during their early differentiation steps in which LIF or MMP1 metalloproteinase are essential. We characterized a differential effect of LIF on mESC-derived cells (either at a committed or differentiated state) and identified *Klf5*, a known pluripotency gene which blocks differentiation towards mesoderm lineage [50,60], as one of the factors controlling this effect. This new property of Klf5 on modulating mESCs plasticity could be in link with its potentiality to reprogram somatic cells, a mechanism favoured by specific miRNAs [61,62] and to its role to govern homeostasis and oncogenesis in stem and transit-amplifying cells of the intestinal epithelia [63].

Other *speLifind* genes, mainly induced by LIF in the reversible window of cell differentiation, could also be essential effectors of plasticity rather than the *PleioLifind* genes and will deserve further investigation.

We observed serum batch-dependent variations in the plasticity window. Indeed serum composition fluctuates from batch to batch, probably including varying amounts of BMP2/BMP4, respectively involved in cardiomyocyte differentiation (BMP2) or maintenance of cell pluripotency in synergy with LIF (BMP4) [52,64]. To avoid the control of foetal calf serum (FCS) parameter for each serum batch change, we performed the plasticity test in the serum replacement medium in which cell differentiation occurs upon LIF withdrawal with less morphological differences than in serum medium (data not shown), but in which differentiated cells undergo apoptosis as previously shown under classical cell growth condition in FCS [65–67]. We observed that the plasticity window was slightly delayed with an effect of LIF extended up to d2 for some genes in the presence of SR containing medium. This is in agreement with a previous study demonstrating that cells in G1 were more prone to differentiate than cells in G2/M. Indeed, in this report it was shown, in a ‘LIF rescue assay’, that LIF regulates the extent of cell cycle phases up to d2 [68].

MMP1 was recently shown to maintain cell pluripotency by releasing trapped cytokine in the ECM. Our results demonstrated that MMP1 maintained also cell plasticity in committed cells up to d2, probably by CNTF delivery, a process which remains to be demonstrated in our assay. Whether ECM remodeling upon MMP1 activity could synergize with LIF during this process, remains also to be determined.

Since the starting population of mESCs is heterogeneous [69–71], there was a possibility that after one day of starvation, LIF addition could favor the clonal expansion of a sub-population of cells which are resistant to LIF withdrawal and remain pluripotent. However, when following protein expression encoded by one of the Pluri gene (*Ceacam1*) by flow cytometry analysis, we did not detect any residual resistant population which could have been observed by additional peak of fluorescence in LIF withdrawal kinetic, in such analysis (Fig 2D). Also, the fact that we observed an induction of *Pluri* genes and a repression of *Diff* genes after one day of treatment (in the -LIFd1+LIFd1 condition, as shown in S1 Fig) argues against a clonal expansion of resistant cells. In addition, in a ‘LIF rescue assay’, it has been shown that LIF reverts the differentiation-dependent increase of G1 phase up to d2 [57]. Moreover, the LIF effect is lost on cells differentiated for two days or more, stressing the point that LIF displays differential effects depending upon cell maturity and that there is a decisive step for commitment between two and three days of LIF withdrawal. Furthermore, EBs grown for more than 20 days *in vitro* can re-express *Oct4* and recover pluripotent properties, presenting another window of potential plasticity in the mESC model [72]. However, it has not been described whether this late effect is LIF-dependent, which could be a possibility since LIF is expressed in differentiated cells from day 6 and later ([42] and our unpublished results).

PI3K regulates developmental and physiologic processes during myogenesis and promotes growth and osteogenic differentiation of rat MSCs upon mechano-growth factor (MGF) activation [73,74]. We showed that the PI3K pathway is involved in mESC-derived cell choices and particularly in the expression of the key Brachyury early mesodermal marker, but not at early times of LIF-dependent plasticity. Since PI3K is known to maintain mESCs pluripotency [16,75], this result indicates that the mechanisms of pluripotency and plasticity, despite having common regulators like *Klf5*, are not similar. Further characterization of this LIF-dependent window of plasticity, by chemicals or siRNA library screenings, will require new tools as tagged cells sorted by gain or loss of a short life fluorescent protein expressed under the control of a LIF-dependent promoter (the *Pluri* genes *Ceacam 1*, *Esrrb* or *early Diff* gene like *Lef1*).

Classical cell growth cultures are far from being ideal, in particular because O_2_ is much lower *in vivo* than in cell culture incubators (20%). However, studies of mESCs aiming at taking this parameter into consideration remain controversial and the outcome of hypoxia is poorly understood. The various O_2_ levels (1 to 5% O_2_) and mESC lines, grown with or without feeders (D3 versus CGR8 for example) could explain differences in published reports. Also, avoiding normoxic shock during medium changes, by using a hypoxia cell chamber, is an important parameter to diminish technical variations in the effect of hypoxia (our unpublished observation). We showed in this study that under hypoxia, mESCs respond poorly to LIF, probably because of low expression of the specific LIF receptor subunit, gp190, as seen both at the RNA and protein levels. We showed that the level of STAT3 repressor, *Socs3*, is not changed between normoxia and hypoxia at the RNA level (data not shown), but we cannot rule out that a stabilized Socs3 protein could be responsible for the low level of phospho-Tyr 705-STAT3 protein detected under hypoxia. We also observed that the level of Oct4, Nanog and Sox2 proteins was reduced under hypoxia compared to normoxia, despite similar RNA expression levels. This indicates hypoxia-dependent translational regulation in agreement with a recent report showing variation in mRNA translation under hypoxia in the adrenal gland phaerochromocytoma PC12 model [76]. However, we showed that cells under hypoxia maintain *in vitro* pluripotent criteria. Indeed, they are able to form EBs that differentiate into the three mature germ layers and to neurons and beating cardiomyocytes. Also, we demonstrated that LIF-dependent cell plasticity is maintained under hypoxic conditions at d1. Whether this reversible state of cells could be maintained under hypoxia at later times after LIF withdrawal remains to be investigated. Though, we speculate that mESCs, which have been derived under 20% O_2_, adapted their potential to remain pluripotent and plastic by expressing *Master* and *Pluri* genes at higher levels than needed under physioxic conditions (3% O_2_). It will be of great interest to perform exhaustive transcriptomic and proteomic analyses in fresh mESCs directly derived under low O_2_ and to compare them with data so far obtained under 20% O_2_. This might lead to characterization of new stemness factors that could operate in hypoxic niches in embryos.

In conclusion, we set up an *in vitro* test allowing to characterize the impact of cytokines (like LIF in this study), of genes (by siRNA strategy), of signalling pathways (by use of chemicals) and of various environmental parameters on mESCs plasticity. We unveiled a new property of *Klf5* to regulate this LIF-dependent effect which is not under strict control of the PI3K pathway. We also show that MMP1 can replace LIF to a certain extent in the plasticity test and that mESCs remain plastic under hypoxic conditions, despite alterations in gene expression patterns.

## Supporting Information

S1 Fig Gene modulation starts within the first 24h of LIF addition. Cells were depleted from LIF or induced with LIF after a period of LIF depletion as indicated. After two days, RNA was extracted and expression of the selected genes was analyzed by RT-qPCR with *Hprt* used for normalization. Mean and SEM (Standard Error of Mean) bars were calculated from 4 independent experiments. +LIF condition was arbitrarily set as 1.
(TIF)

S2 Fig Experiments in serum replacement versus FCS-containing medium slightly modulates the plasticity window. Cells were depleted from LIF or induced with LIF after a period of LIF depletion as indicated. After 4 days, expression of the selected genes (A) Master genes, (B) *Pluri* genes and (C) *Diff* genes was analyzed. Graphs represent the average level of expression and SEM as depicted in Fig 2. One sample t-test was performed for each condition *versus* the +LIF sample: *p-value<0.05; **p-value<0.01, ***p-value<0.001; if not stated: not significant.
(TIF)

S3 Fig Quantification of Western blot experiments. Graph represents the mean of ratio of normoxia versus hypoxia signals obtained in the +LIF condition for each antibody, as indicated, with normalization performed with the ERK2 protein as a loading control. n = 4. Quantification was performed with the Odyssey ^FC^ (LI-COR) quantification Image Studio software.
(TIF)

S4 Fig mESCs maintain alkaline phosphatase activity and mESC-like morphology under hypoxia. Pictures of mESCs grown with LIF under normoxia or hypoxia for four days and stained with the Alkaline phosphatase kit (Sigma-Aldrich, 86R-1KT). Scale bar is 100 μM.
(TIF)

S5 Fig List of primers used for RT-qPCR. (DOCX)
