# Supplementary figures and images for: Murine Embryonic Stem Cell Plasticity Is Regulated through Klf5 and Maintained by Metalloproteinase MMP1 and Hypoxia
Source: PLoS One. 2016 Jan 5;11(1):e0146281. doi: 10.1371/journal.pone.0146281 (PMC4701481; doi:10.1371/journal.pone.0146281)

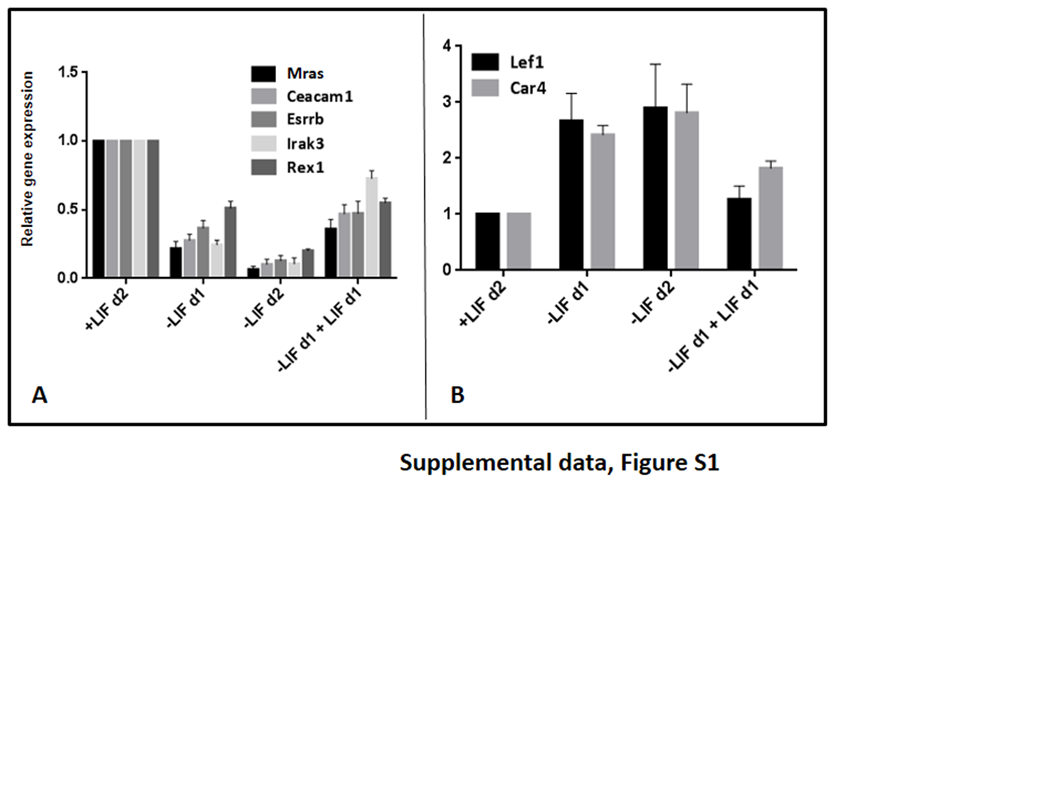

Supplement: S1 Fig — Cells were depleted from LIF or induced with LIF after a period of LIF depletion as indicated. After two days, RNA was extracted and expression of the selected genes was analyzed by RT-qPCR with Hprt used for normalization. Mean and SEM (Standard Error of Mean) bars were calculated from 4 independent experiments. +LIF condition was arbitrarily set as 1. (TIF) [file pone.0146281.s001.tif]

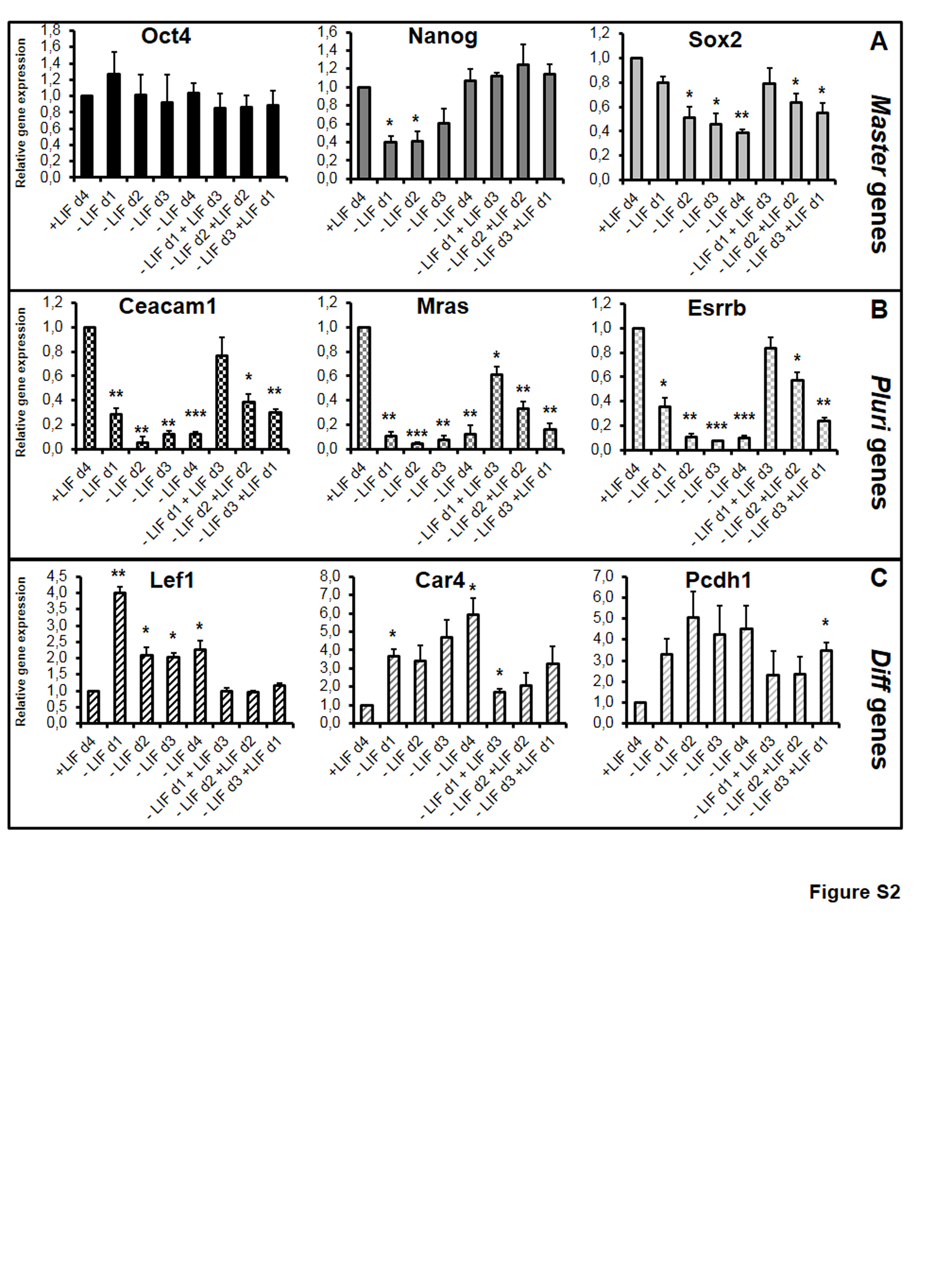

Supplement: S2 Fig — Cells were depleted from LIF or induced with LIF after a period of LIF depletion as indicated. After 4 days, expression of the selected genes (A) Master genes, (B) Pluri genes and (C) Diff genes was analyzed. Graphs represent the average level of expression and SEM as depicted in Fig 2. One sample t-test was performed for each condition versus the +LIF sample: *p-value<0.05; **p-value<0.01, ***p-value<0.001; if not stated: not significant. (TIF) [file pone.0146281.s002.tif]

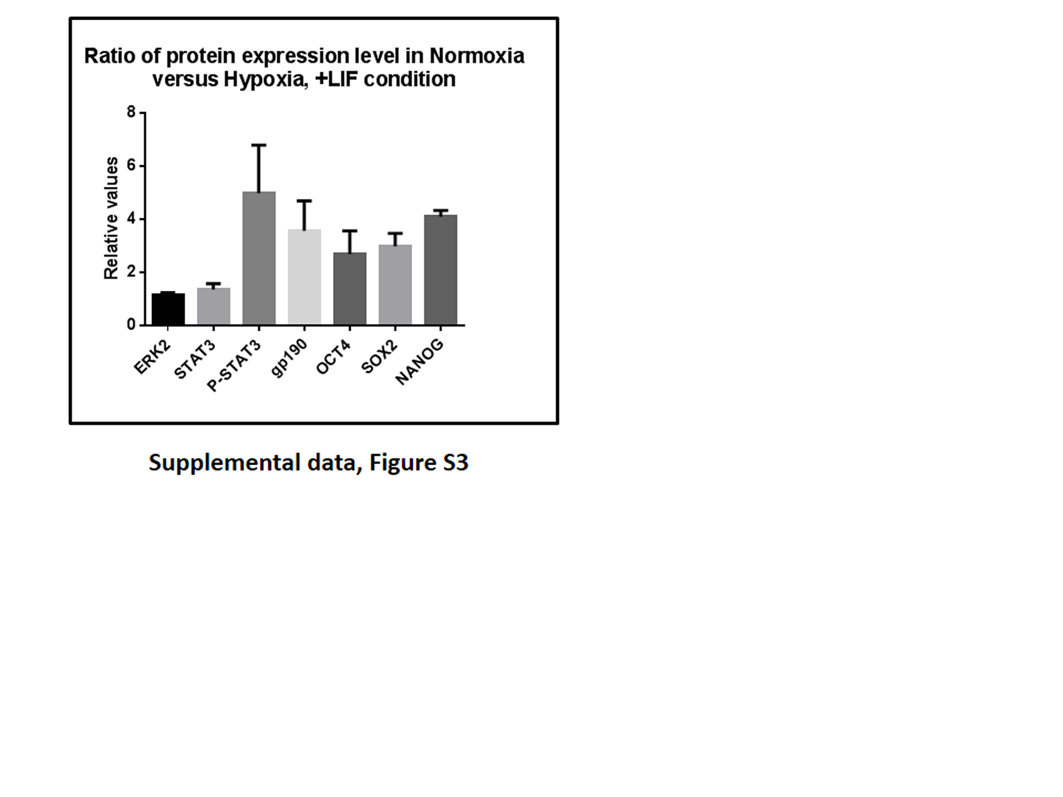

Supplement: S3 Fig — Graph represents the mean of ratio of normoxia versus hypoxia signals obtained in the +LIF condition for each antibody, as indicated, with normalization performed with the ERK2 protein as a loading control. n = 4. Quantification was performed with the Odyssey FC (LI-COR) quantification Image Studio software. (TIF) [file pone.0146281.s003.tif]

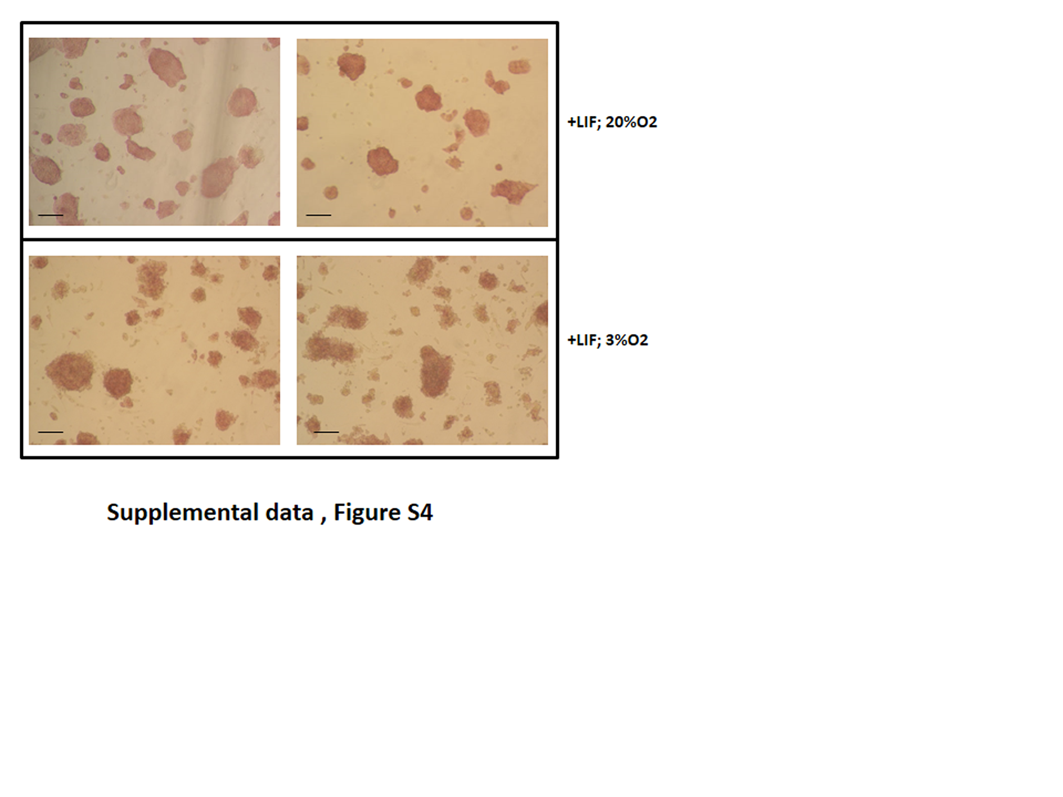

Supplement: S4 Fig — Pictures of mESCs grown with LIF under normoxia or hypoxia for four days and stained with the Alkaline phosphatase kit (Sigma-Aldrich, 86R-1KT). Scale bar is 100 μM. (TIF) [file pone.0146281.s004.tif]
