# Supplementary material for: Murine Embryonic Stem Cell Plasticity Is Regulated through Klf5 and Maintained by Metalloproteinase MMP1 and Hypoxia
Source: PLoS One. 2016 Jan 5;11(1):e0146281. doi: 10.1371/journal.pone.0146281 (PMC4701481; doi:10.1371/journal.pone.0146281)
Supplement: S5 Fig — (DOCX) [file pone.0146281.s005.docx]

**S5 Fig: List of primers used for RT-qPCR experiments**

|  | F: Forward: 5' |  | Size |
| --- | --- | --- | --- |
|  | R: Reverse: 5' |  | amplicon |
|  |  |  |  |
| nm_013556 | Hprt F | tcagtcaacgggggacataaa | 142 |
|  | Hprt R | ggggctgtactgcttaaccag |  |
|  |  |  |  |
| nm_013633 | Oct4 F | gaggagtcccaggacatgaa | 153 |
|  | Oct4 R | agatggtggtctggctgaac |  |
|  |  |  |  |
| nm_011443 | Sox2 F | gcggagtggaaacttttgtcc | 156 |
|  | Sox2 R | gggaagcgtgtacttatccttct |  |
|  |  |  |  |
| nm_028016 | Nanog F | tcttcctggtccccacagttt | 100 |
|  | Nanog R | gcaagaatagttctcgggatgaa |  |
|  |  |  |  |
| nm_011934 | Esrrb F | acacttggggaccagatgag | 150 |
|  | Esrrb R | cctaccaggcgagagtgttc |  |
|  |  |  |  |
| X15351 | Ceacam 1 F | actatcgtcgtactcaggcga | 98 |
|  | Ceacam 1 R | gtcaccctccacgggattg |  |
|  |  |  |  |
| nm_008624 | Mras F | tgttccaagtgaaaaccttccc | 117 |
|  | Mras R | gggtcgtagtcaggcacaa |  |
|  |  |  |  |
| nm_010703 | Lef1 F | aagaaatgagagagcgaatgtcgt | 244 |
|  | Lef1 R | ttctgggacctgtacctgaagt |  |
|  |  |  |  |
| nm_007607 | Car4 F | gacaacggttcagagcacag | 155 |
|  | Car4 R | aagcccttgttcaccttgtc |  |
|  |  |  |  |
| nm_016701 | Nestin F | agaatgtgcagtcaccaagg | 138 |
|  | Nestin R | gggtctcattttcaggtgggt |  |
|  |  |  |  |
| nm_011441 | Sox17 F | GATGCGGGATACGCCAGTG | 136 |
|  | Sox17 R | ccaccacctcgcctttcac |  |
|  |  |  |  |

nm_029357 Pcdh1 F GCTCCGTATCTTCGAGTGGAT 97

Pcdh1 R GGATCTGGTTCTGGCATTCAC

nm_009769 Klf5F ACCTTACAGCATCAACATGAACG 123

Klf5R TGGCTGAAAATGGTAACAGGTT

nm_009337 Tcl1F GTGTACTTGGATGAGTTTCGTCG 153

Tcl1R TTGCCACATTAAAGGCAGCTC

nm_008416 JunBF CCTTGAGACCCCGATAGGGA 260

JunBR TCACGACGACTCTTACGCAG

nm_ 008092 Gata4F CCCTACCCAGCCTACATGG 139

Gata4R ACATATCGAGATTGGGGTGTCT

nm_011441 BrachyuryF GCTTCAAGGAGCTAACTAACGAG 117

BrachyuryR CCAGCAAGAAAGAGTACATGGGC

nm_013584 Gp190F ACCTCCTCCTTACTACTGAAGTG 102

Gp190R TTCCGTCCTTGGATTCTGTAGA

nm_010560 Gp130F CCGTGTGGTTACATCTACCCT 180

Gp130R CGTGGTTCTGTTGATGACAGTG

NC_000074 Tuj1F (Tubb3) CCATCTAGCTACTGACACTG 91

Tuj1R AAGTGGACTCACATGGAGTG

NC_000077 GfapF AGAAAACCGCATCACCATTC 262

GfapR TCACATCACCACGTCCTTG

nm_001170537 Mef2cF GTCAGTTGGGAGCTTGCACTA 112

Mef2cR CGGTCTCTAGGAGGAGAAACA

NC-000074 Rex1F CCCTCGACAGACTGACCCTAA 100

Rex1R TCGGGGCTAATCTCACTTTCAT

nm_010291 Gjb5F CCAAGCCCTCCGAGAAAAACA 107

Gjb5R ACACCGCTTAATCACTAGGTAGA

nm_023844 Jam2F GTGCCCACTTCTGTTATGACTG 113

Jam2R TTCCCTAGCAAACTTGTGCCA

nm_010203 Fgf5F GAAAAGACAGGCCGAGAGTG 102

Fgf5R GAAGTGGGTGGAGACGTGTT

nm_009760.4 Bnip3F TCCTGGGTAGAACTGCACTTC 83

Bnip3R GCTGGGCATCCAACAGTATTT

AC_000026 Glut1F CAGTTCGGCTATAACACTGGTG 156

Glut1R GCCCCCGACAGAGAAGATG

15030115a1 Ncam1F AGCGCAGGTGCAGTTTGAT 269

Ncam1R ACAAAGAGCTTTTACGGACTGG

### 26327895a1 Kdr1F AGTCTACAGTGTGTCAGAAGGG 176

Kdr1R TGCAAAGTCCTTGACGTTCAC

31982513a1 Afp F CTTCCCTCATCCTCCTGCTAC 145

Afp R ACAAACTGGGTAAAGGTGATGG
